# Ubiquitous neurocognitive dysfunction in familial adenomatous polyposis: proof-of-concept of the role of APC protein in neurocognitive function

**DOI:** 10.1186/s13053-020-0135-3

**Published:** 2020-02-24

**Authors:** Marcia Roxana Cruz-Correa, Ana Cecilia Sala, Beatriz Cintrón, Jessica Hernández, Myrta Olivera, Adrian Cora, Constance M. Moore, Carlos A. Luciano, Marievelisse Soto-Salgado, Francis M. Giardiello, Stephen R. Hooper

**Affiliations:** 1 Department of Medicine, University of Puerto Rico School of Medicine, UPR Medical Sciences Campus, PO BOX 365067, San Juan, 00936 Puerto Rico; 2 Department of Biochemistry, University of Puerto Rico School of Medicine, San Juan, Puerto Rico; 3 Department of Medicine, Neurology Section, University of Puerto Rico School of Medicine, San Juan, Puerto Rico; 4 Division of Cancer Biology, University of Puerto Rico Comprehensive Cancer Center, San Juan, Puerto Rico; 5 Division of Gastroenterology, School of Medicine, Johns Hopkins University, Baltimore, MD USA; 6 University of Massachusetts Medical School, Worcester, MA USA; 7 Department of Allied Health Sciences, School of Medicine, University of North Carolina-Chapel Hill, Chapel Hill, NC USA

**Keywords:** Familial adenomatous polyposis, Neurocognition, Hispanics, FAP

## Abstract

**Background:**

Familial adenomatous polyposis (FAP) is an autosomal dominant disorder caused by germline mutations in the APC gene. Patients with FAP have multiple extraintestinal manifestations that follow a genotype-phenotype pattern; however, few data exist characterizing their cognitive abilities. Given the role of the APC protein in development of the central nervous system, we hypothesized that patients with FAP would show differences in cognitive functioning compared to controls.

**Methods:**

Matched case-control study designed to evaluate cognitive function using the Test of Nonverbal Intelligence-4, the Bateria III Woodcock-Munoz, and the Behavior Rating Inventory of Executive Functions-Adult. Twenty-six individuals with FAP (mean age = 34.2 ± 15.0 years) and 25 age-gender and educational level matched controls (mean age = 32.7 ± 13.8 years) were evaluated.

**Results:**

FAP-cases had significantly lower IQ (*p* = 0.005). Across all tasks of the Batería III Woodcock-Muñoz, FAP-cases performed significantly lower than controls, with all of the summary scores falling in the bottom quartile compared to controls (*p* < 0.0001). Patients with FAP scored within the deficient range for Long-Term Retrieval and Cognitive Fluency.

**Conclusion:**

APC protein has an important role in neurocognitive function. The pervasive nature of the observed cognitive dysfunction suggests that loss or dysfunction of the APC protein impacts processes in cortical and subcortical brain regions. Additional studies examining larger ethnically diverse cohorts with FAP are warranted.

## Introduction

Familial adenomatous polyposis (FAP) is an autosomal dominant disorder caused by germline mutations in the Adenomatous Polyposis Coli (APC). There are multiple extraintestinal manifestations that follow a genotype-phenotype pattern [1]; however, few data characterize the cognitive abilities of individuals with FAP. The APC protein has a major function in the development of the central nervous system, including a pivotal role in the development of the pre-post synaptic complex seen in *in-vivo* [2] and animal studies [3]. Moreover, high concentrations of the APC protein are present in the hippocampus, thalamus, and limbic system. Consequently, specific difficulties in the neurocognitive domains of attention, memory, and executive functions among individuals with deficient APC protein function might be expected.

Despite these known linkages few empirical findings examining the relationship between the expression of the APC protein and neurocognitive and behavioral functioning in individuals with APC mutations exist [4–7]. To date, animal studies have associated APC dysfunction with altered synaptic function in APC cKO mice, which plays a critical role in learning and memory [8]. The genetic changes in APC that have been implicated in learning and memory deficits, repetitive behaviors, and reduced social interest [8], also have been associated with working memory deficits, increased locomotion, and anxiety-related behavior [3]. Though frequently hypothesized that genetic factors play an important role in psychiatric disorders, identification of specific genes has been a difficult task. Interestingly, preliminary findings implicate the APC gene in psychiatric disorders such as schizophrenia [9, 10] and depression [11]. These psychiatric conditions have also been related to cognitive and behavioral impairments.

The primary aims of this exploratory study were to determine: (1) whether patients with FAP would evidence differences in performance on measures of neurocognitive function compared with non-affected controls; and (2) if neurocognitive manifestations followed a genotype-phenotype distribution as seen with other clinical manifestations in FAP. *Given the major role of the APC protein in the development of the central nervous system*, including the development of the pre-post synaptic complex seen in *in vitro* [2, 12] and *in vivo* [8] studies, we hypothesized that individuals with FAP would show lower cognitive functioning compared to non-FAP controls across all ages. Given the potential for synaptic dysfunction, we would expect tasks related to processing speed and cognitive efficiency to be the most affected.

## Methods

### Participants

Sample size calculations were based on data from O’Malley et al. [13] who reported a lower Verbal IQ for individuals with FAP compared to historical average scores. Using the Batería III Woodcock-Muñoz, with a mean IQ = 100 (SD = 15) a sample size of 23 in each group will have 90% power to detect a probability of 0.240 (effect size) that an observation in Group 1 is less than an observation in Group 2 using a Wilcoxon (Mann-Whitney) rank-sum test with a 0.050 two-sided significance level. Participants for both groups (ages ≥10 years) were ascertained via the Puerto Rico Familial Colorectal Cancer Registry (http://purificar.rcm.upr.edu/index_eng.html).

Inclusion criteria for cases included genetically confirmed FAP (based on a mutation on the APC gene performed by commercial laboratory testing), age ≥ 10 years, able to assent (children) and consent (adults) to participate in this study, and able to complete all neurocognitive testing. Inclusion criteria for controls included no known family history of FAP, negative clinical diagnosis of FAP based on colonoscopy (for adults), and willingness to undergo neurocognitive testing. Participants were all Spanish-speaking individuals from Puerto Rico. As education and age are correlated with IQ, cases were matched to non-FAP control individuals with regards to age (± three years), gender, and education (less than high school, at least high school, bachelors-degree or post-graduate education). Exclusion criteria for both cases and controls included previous diagnosis of any major psychiatric condition given the potential impact of these conditions on neurocognitive functioning, inability to sign/assent/consent study participation, or complete the neurocognitive tests.

Participants signed informed consent (or assent for children) prior to participation in the study and were evaluated at the Puerto Rico Clinical and Translational Research Consortium at the University of Puerto Rico (UPR) Medical Sciences Campus. The research protocol was approved by the Institutional Review Board of the UPR Medical Sciences Campus.

#### Measures and procedures

Neurocognitive procedures were carried out by licensed psychologists with extensive expertise in evaluation of neurocognitive disorders in children with genetic conditions. Selection of measures for this study was based on the following criteria: (1) tests have hypothesized links to brain regions where there are known high concentrations of APC protein; (2) age-appropriateness, with tasks going across the lifespan; (3) well established standardization and age-based normative data; and (4) strong psychometric properties. These outcome measures included the Test of Nonverbal Intelligence-4, the Batería III Woodcock-Mũnoz, and the Behavior Rating Inventory of Executive Functions-Adult. Measures of anxiety (General Anxiety Disorder Questionnaire-7), social communication (Social Communication Questionnaire), and depression (Patient Health Questionnaire-9) were used as potential covariates.

##### Batería III Woodcock-Mũnoz

We used the cognitive components of this battery, which was translated and adapted from the publisher of the Woodcock-Johnson III Cognitive Battery via a calibration sample of approximately 1400 individuals from both the United States and several Spanish speaking countries including Puerto Rico. This battery is based on the Cattell-Horn-Carroll model of cognitive abilities and includes measures of comprehension-knowledge, visual-spatial thinking, auditory processing, processing speed, short-term memory, long-term memory, working memory, broad attention, and executive processes. This measure retains a high degree of technical adequacy with reliability for all of the clusters being ≥0.90 across all of the age ranges.

##### Test of nonverbal intelligence (TONI-4)

Although the Batería III Woodcock-Mũnoz can generate a General Intellectual Factor, its use as a covariate in any of our analyses will confound group findings from the cognitive clusters as the intellectual estimate contains many of the same subtests. In that regard, for our overall measure of intellectual functioning, we have included a nonverbal measure of intellectual functioning: the TONI-4. While hoping to utilize the KBIT-2 to replicate the findings of O’Malley et al. [13] there is no Spanish version of this test; consequently, using the TONI-4 provides a broader application for our sample and serves as a subject descriptive variable and as a potential covariate in the data analyses. The TONI-4 measures the ability to solve problems without overtly using language or complex motor responses, requiring the person to apply reasoning strategies to a problem defined by novel abstract figures. It includes verbal directions in Spanish, and responses require only simple pointing or gestures. The psychometric properties and normative basis of the TONI-4 are strong.

##### Behavior rating inventory of executive functions (BRIEF)-parent and adult versions

The BRIEF is a behavior rating scale of executive functions in everyday life that is completed by the parent (up to age 18 years) or an individual (ages 18 and older). The BRIEF provides ecological measures of a variety of executive functions that address regulatory activities in day-to-day functioning. It comprises specific clinical scales (e.g., Organization, Shift, Working Memory, etc.) and three summary scales: Behavioral Regulation Index, Metacognition Index, and Global Executive Composite. Each version of the scale is standardized, well normed, has strong psychometric properties, and is available in both English and Spanish.

##### Patient health questionnaire (PHQ9)

The PHQ9 is a nine-item depression scale completed by the individual. It is a multiple-choice self-report inventory used as a screening and diagnostic tool for depression, anxiety, alcohol, eating, and somatoform conditions.

##### Generalized anxiety disorder (GAD-7)

The GAD-7 is a self-reported questionnaire for screening and severity of generalized anxiety disorder. The GAD-7 has seven items, which measure severity of various signs of generalized anxiety disorder according to reported response categories with assigned points.

#### Neurological exam

All patients underwent a full neurologic examination exam performed by a neurologist that included a screening for mental status, apraxia, agnosia, aphasia, cranial nerve status, motor and sensory intactness, motor coordination, and deep tendon and corticospinal tract reflexes.

#### Data analyses

Preliminary data analyses included a comparison of selected sociodemographic characteristics by study group (FAP vs. Control) using χ^2^ test or Fisher’s exact test. The anxiety and depression scores were compared by study group (FAP vs. Control) using t-tests or Wilcoxon rank-sum test. To address group differences in neurocognition, simple and multiple linear regression models were used to estimate the unadjusted and adjusted beta (B) coefficients with their 95% confidence intervals. The models were adjusted by age, gender, and educational level. *P*-values were corrected using the Bonferroni-Holm method (https://www.statisticshowto.datasciencecentral.com/holm-bonferroni-method/). To establish significance, observed *p*-values were compared with corrected *p*-values. If (1) observed *p*-values were lower than corrected *p*-values, and (2) there was an intersection between the significant *p*-values across the tests for each measure, then, neurocognitive measure was established to be significant.

For the evaluation of neurocognitive function status across the APC gene mutation spectrum, FAP patients were stratified according to location of mutations in the gene. Specifically, genotype-phenotype analysis was performed comparing individuals with mutations in codons within the attenuated-FAP region (codons < 1000 and > 1500; *N* = 9) and those within the mutation cluster region (between codons 1000 to 1500; *N* = 17). To determine the effect of the FAP genotype-phenotype, we used simple linear regression models and adjusted linear regression models with 95% confidence intervals. The models were adjusted by age, gender, and educational attainment. Statistical analyses were performed using Stata 14.0.

## Results

### Baseline demographic and clinical characteristics

Baseline demographic characteristics are presented in Table 1. Study participants (cases and controls) were equivalent with regards to age (mean age 34.2 ± 15.0 vs. 32.7 ± 13.8, *p* = 0.59), gender (*p* = 0.68), and educational level (*p* = 0.10), with both groups being unremarkable in the reporting of their symptoms. There were no statistically significant differences between patients with FAP and controls with regard to baseline anxiety (*p* = 0.21) and depression (*p* = 0.09) (data not shown).

**Table 1:** Baseline demographic characteristics of study participants

Characteristic	FAP group (*n* = 26)	Control group (*n* = 25)	*p*-value*
Age			0.59
< 21 years	8 (30.8)	6 (24.0)	
≥ 21 years	18 (69.2)	19 (76.0)	
Mean ± SD	34.2 ± 15.0	32.7 ± 13.8	
Gender			0.68
Male	14 (53.9)	12 (48.0)	
Female	12 (46.2)	13 (52.0)	
Medical insurance			0.28
None	1 (4.2)	2 (8.3)	
Private	13 (54.1)	17 (70.8)	
Public	10 (41.7)	5 (20.8)	
Educational level			0.09
< 12	4 (15.4)	9 (36.0)	
≥ 12	22 (84.6)	16 (64.0)	
Grade repetitions			0.45
No	15 (57.7)	17 (68.0)	
Yes	11 (42.3)	8 (32.0)	

*p-value from the χ^2^ test or Fisher’s exact test

### Intelligence

Group differences on the TONI-4, our measure of Intelligence, are presented in Table 2. Both FAP cases and controls fell within the average range of functioning, although the FAP patients had statistically significantly lower functioning compared with age-, gender- and education-matched controls (90.5 ± 5.6 vs. 95.6 ± 6.8, *p* = 0.008).

**Table 2:** Comparison of the FAP and control groups on the neurocognitive functions

Measure	FAP group (n = 26)		Control group (n = 25)		*p*-value*	Simple LRM (coefficients)	*p*-value	Adjusted multivariate LRM** (coefficients)	*p*-value	Adjusted multivariate LRM*** (coefficients)	*p*-value
	Mean	SD	Mean	SD							
TONI-4 IQ	90.5	5.6	95.6	6.8	0.005	−5.1	0.005	−5.2	0.005	−5.0	0.008
Batería III clusters											
Verbal ability^#^	88.2	7.7	95.7	7.8	0.001	−7.5	0.001	−7.4	0.001	−7.2	0.002
Thinking ability^#^	80.5	10.5	97.8	10.3	< 0.0001	−17.3	< 0.0001	−16.7	< 0.0001	− 16.9	< 0.0001
Cognitive efficiency^#^	81.1	11.9	94.7	9.9	0.0001	−13.6	< 0.0001	− 13.4	< 0.0001	− 13.3	< 0.0001
Long-term memory^#^	66.8	14.3	85.1	12.1	< 0.0001	−18.3	< 0.0001	−17.8	< 0.0001	− 18.3	< 0.0001
Processing speed^#^	85.1	14.7	102.0	9.7	< 0.0001	−16.9	< 0.0001	− 16.6	< 0.0001	− 17.2	< 0.0001
Phonemic awareness	88.6	10.8	97.6	8.7	0.002	−8.9	0.002	−8.7	0.002	−8.5	0.004
Working memory^#^	84.4	10.7	95.6	11.4	0.0007	− 11.1	0.001	− 11.2	0.001	−11.2	0.001
Cognitive fluency^#^	78.3	12.2	91.8	9.9	0.0001	−13.5	< 0.0001	− 13.5	< 0.0001	− 14.8	< 0.0001
Executive processes^#^	83.8	13.7	100.8	9.7	< 0.0001	−17.0	< 0.0001	− 17.0	< 0.0001	− 17.1	< 0.0001
BRIEF-Adult											
Inhibit	49.3	7.9	47.1	7.0	0.319	2.3	0.319	2.4	0.305	2.0	0.385
Shift	51.8	10.3	48.9	11.9	0.256	2.9	0.391	3.2	0.391	2.9	0.396
Emotional control	51.5	8.6	47.7	10.2	0.067	3.9	0.180	4.0	0.162	3.6	0.225
Self-monitor	49.5	8.6	46.4	9.8	0.212	3.1	0.270	3.2	0.251	3.1	0.291
Initiate	50.5	10.9	49.2	9.4	0.795	1.3	0.677	1.5	0.626	1.5	0.642
Working memory	53.7	11.5	49.4	8.9	0.218	4.3	0.178	4.6	0.112	4.4	0.180
Plan/Organize	50.3	10.9	49.0	13.9	0.587	1.3	0.731	1.7	0.622	1.1	0.772
Task monitor	51.5	11.5	48.7	8.7	0.571	2.8	0.372	3.2	0.240	2.4	0.452
Organization of materials	49.8	8.2	50.3	8.7	0.870	−0.4	0.870	−0.2	0.929	−0.7	0.779
Behavior regulation index	50.8	8.8	47.6	10.5	0.266	3.3	0.266	3.5	0.214	3.3	0.278
Metacognitive index	51.1	10.8	49.2	9.8	0.595	1.9	0.549	2.2	0.441	1.7	0.589
Global executive composite	51.1	10.0	48.3	10.1	0.272	2.8	0.356	3.1	0.264	2.7	0.389

*p-values from the T-test or Wilcoxon rank-sum (Mann-Whitney) test; **Adjusted by age and gender; ***Adjusted by education. *LRM* Linear Regression Models. ^#^Neurocognitive measures are significant under the Bonferroni-Holmes corrections for all the statistical tests simultaneously

### Neurocognitive functioning

#### Batería-III summary scores

Findings of the groups on the Batería-III summary scores are shown in Table 2. Across all of the Batería-III summary scores, FAP cases reflected *significantly* more risk for cognitive dysfunction than the controls, with the cases showing performances in the lower quartile on Verbal Ability, Thinking Ability, Cognitive Efficiency, Long-Term Retrieval, Processing Speed, Phonemic Awareness, Working Memory, Cognitive Fluency, and Executive Processes. In particular, FAP cases scored within the deficient range for Long-Term Retrieval and Cognitive Fluency.

Specific group comparisons on the obtained scores revealed a similar pattern of findings wherein the FAP Group performed significantly lower than controls on: Verbal Ability (LRM − 7.2, *p* = 0.002); Thinking Ability (LRM − 16.9, *p* < 0.0001; Cognitive Efficiency (LRM − 13.3, p < 0.0001); Long-Term Memory (LRM − 18.3, p < 0.0001); Processing Speed (LRM − 17.2, p < 0.0001; Phonemic Awareness (LRM − 8.5, *p* = 0.004); Working Memory (LRM − 11.2, *p* = 0.001); Cognitive Fluency (LRM − 14.8, p < 0.0001); and Executive Processes (LRM − 17.1, p < 0.0001).

#### Batería-III subtests

An exploratory examination of group differences on the Batería-III subtests was conducted to determine if any of the subtests were influential in the summary score findings. Specifically, 13 of the 15 subtests produced significant group differences, with FAP cases performing below controls on each of these 13 subtests. Effect sizes were in the large range in every instance. After Bonferonni Correction (i.e., *p* < 0.003), nine of the Batería-III subtests remained statistically significant including: Verbal Comprehension, Visual-Auditory Learning, Spatial Relations, Sound Blending, Concept Formation, Visual Matching, Auditory Working Memory, Retrieval Fluency, and Decision Speed.

### Group differences in ratings of executive functions

We only had five cases where parents and child needed to complete the BRIEF, and all fell within the average range. Consequently, given the small sample size, no further analysis was conducted (data not shown). For the Adult BRIEF, both cases and controls rated their executive functioning to be within the average range. Group comparisons (FAP cases vs. controls) did not show any significant differences on any of the individual or summary scales of the BRIEF-Adult (Table 2).

### Genotype-phenotype correlation analysis

Neurocognitive status was analyzed according to APC mutation location (Table 3). FAP cases were grouped in two categories based on APC gene mutations: attenuated FAP (mutations in codons < 1000 and > 1500; *N* = 9) and classic FAP, those with an APC gene mutation in the mutation cluster region (between codons 1000 to 1500; *N* = 17). The two groups did not differ on age or gender (*p* > 0.05), respectively (data not shown). Findings revealed that the two FAP groups were not significantly different on IQ (*p* = 0.709). However, FAP cases with mutations located within the classic mutation cluster had marginally lower performance on Woodcock-Muñoz summary scores of Cognitive Efficiency (*p* = 0.076) and Processing Speed, (*p* = 0.056), and on BRIEF-Adult scales of Shift (*p* = 0.058) and Initiate (*p* = 0.081) compared to FAP cases with mutations in the attenuated FAP region (Table 3).

**Table 3:** Comparisons of FAP Severity Groups on Neurocognitive Functions

Measure	Attenuated FAP Group (*n* = 9)		Classic FAP Group (*n* = 17)		*p*-value*	Simple LRM(coefficients)	*p*-value	Adjusted multivariate LRM** (coefficients)	*p*-value	Adjusted multivariate LRM*** (coefficients)	*p*-value
	Mean	SD	Mean	SD							
TONI-4 IQ	91.2	4.5	90.2	6.3	0.662	−1.0	0.662	−1.0	0.670	−0.9	0.709
Batería III clusters											
Verbal ability	89.7	5.5	87.4	8.7	0.479	−2.3	0.479	−2.3	0.504	−2.0	0.566
Thinking ability	81.8	10.0	79.9	10.9	0.669	−1.9	0.669	−2.0	0.671	−1.8	0.704
Cognitive efficiency	87.2	6.4	77.8	13.0	0.054	−9.4	0.054	−9.4	0.064	−9.2	0.076
Long-term memory	65.0	16.2	67.7	13.6	0.655	2.7	0.655	2.4	0.677	2.6	0.666
Processing speed	92.9	7.9	81.0	16.0	0.048	−11.9	0.048	−12.1	0.045	−11.4	0.056
Phonemic awareness	91.1	11.1	87.3	10.7	0.400	−3.8	0.400	−3.8	0.419	−4.2	0.379
Working memory	88.6	6.6	82.2	11.9	0.154	−6.3	0.154	−6.2	0.168	−6.1	0.189
Cognitive fluency	82.4	11.0	76.1	12.5	0.210	−6.4	0.210	−6.5	0.164	−6.2	0.193
Executive processes	84.8	10.8	83.2	15.3	0.791	−1.5	0.791	−1.6	0.781	−1.0	0.862
BRIEF-Adult											
Inhibit	50.4	5.9	48.8	8.8	0.657	−1.6	0.657	−1.3	0.712	−0.5	0.886
Shift	57.1	8.1	49.1	10.4	0.052	−8.1	0.068	−7.6	0.055	−7.9	0.058
Emotional control	52.5	6.1	51.0	9.8	0.519	−1.5	0.696	−1.2	0.742	−1.3	0.726
Self-monitor	52.4	9.3	48.9	8.1	0.229	−4.4	0.249	−4.0	0.253	−5.2	0.134
Initiate	55.8	12.2	47.9	9.5	0.070	−7.9	0.095	−7.4	0.082	−7.9	0.081
Working memory	52.9	12.6	54.1	11.4	0.735	1.2	0.818	1.8	0.663	1.3	0.760
Plan/Organize	54.3	12.3	48.3	9.9	0.176	−5.9	0.214	−5.3	0.158	−5.5	0.162
Task monitor	54.8	10.9	49.9	11.8	0.310	−4.8	0.345	−4.1	0.287	−3.6	0.369
Organization of materials	50.8	8.6	49.4	8.2	0.706	−1.4	0.706	−1.1	0.744	−0.5	0.882
Behavior regulation index	53.6	6.7	49.4	9.6	0.281	−4.2	0.281	−3.7	0.270	−3.8	0.283
Metacognitive index	53.9	11.5	49.7	10.5	0.443	−4.2	0.380	−3.6	0.344	−3.6	0.361
Global executive composite	54.1	9.2	49.6	10.2	0.297	−4.6	0.300	−4.0	0.253	−4.0	0.276

*p-values from T-test or Wilcoxon rank-sum (Mann-Whitney) test; **Adjusted by age and gender; ***Adjusted by age, gender and education

### Neurological examination

A full neurologic examination was performed in all 26 cases and in 24 controls. No consistent pattern of abnormalities was observed in the cases or control subjects. Four cases showed findings consistent with a mild distal sensory neuropathy, in three of the cases this was related to prior chemotherapy for colon cancer.

## Discussion

The findings of this study provide a proof-of-concept for an important role of the APC protein in neurocognitive function. Moreover, this investigation is the first to systematically evaluate neurocognition in the FAP population using a carefully matched case-control design, and evaluating important potential confounders such as anxiety, depression, and level of education. Our findings reflected significantly lower performance on IQ and in a variety of neurocognitive functions, with effect sizes being within the large range. Furthermore, for many of the specific neurocognitive functions, individuals with FAP performed in the bottom quartile, suggesting possible difficulties in these areas. Interestingly, there were no group differences noted on the adult self-ratings (BRIEF questionnaire) of executive functions, perhaps secondary to self-perceptions not recognizing the subtle differences that might be present when compared to their peers.

Original observations describing intellectual disabilities among individuals with FAP were limited to case reports [4, 5, 7]. Patients with FAP were shown to perform lower on test of intellectual capacity, particularly on verbal tasks, when compared to patients with ulcerative colitis [14]. In addition, a conference presentation by O’Malley et al. [13] directly examined the cognitive functioning of individuals with FAP by conducting a brief intelligence test with 44 adult subjects from 42 families. They found that the group performed in the average IQ range, with 27% of the sample performing in the bottom quartile. Verbal abilities were significantly lower than average, although not impaired (Verbal IQ = 95.5 ± 12), with the majority of participants having their verbal abilities significantly lower than their nonverbal abilities (*p* = 0.020). Our nonverval abilities were similar in the level to that obtained by O’Malley et al. The authors hypothesize that the APC protein was critical for dependent pathways in the cochlea and could have a role in (verbal) cognition. In contrast, our investigation is the first to incorporate an age-, gender-, and education-matched control group with a complete battery of neurocognitive tests enabling interrogation of multiple domains and corresponding anatomical regions. Moreover, our scientific rationale differs from that of previous investigators as it relates to the *central role of the APC protein in the development of the synaptic complex* in the central nervous system [2, 3].

The APC protein has multiple domains and binding patterns, which indicates a multi-functional nature [15]. The classic colorectal adenomatous polyposis seen in patients with FAP results from the known negative regulation of APC protein in the *Wnt* transcription pathway [16]. The APC protein also carries out microtubule-organizing functions in neurons [17–19], and has a role in synapse assembly [20]. The APC protein is found in many cell types including neurons in the adult rodent brain [21], and high levels of expression of the APC mRNA are observed during brain development [22, 23]. Moreover, APC protein functions in localizing nicotinic acetylcholine receptors (nAChRs) at neuronal synapses *in vivo* [24] organizing a multi-molecular complex that stabilizes the local cytoskeleton and links the alpha-3nAChRs subunit to APC at postsynaptic sites [2]. This complex of proteins is required for coordinating presynaptic and postsynaptic maturation in cholinoceptive neurons in vertebrate animals [24]. This complex is vital in processing information as nicotinic receptors have a key role in attention, learning, and memory and are highly concentrated in the cerebral cortex, hippocampus, thalamus and limbic system [25]. Thus, truncated or absence of the APC protein in the brain among patients with FAP may result in altered neurocognitive functions including attention deficits and learning impairments, and our findings provide preliminary support for these assertions. Given the pathophysiology inherent in these processes, we had hypothesized that patients with FAP would show lower processing speed and cognitive efficiency than controls and, in fact, this is what was uncovered. This was in addition to a significant lower performance than controls in a host of other neurocognitive domains.

In an elegant study using a conditional APC knockout mouse with deletion targeted predominantly to forebrain excitatory neurons (hippocampus and cortex), Mohn et al. reported impaired hippocampal-dependent spatial learning [8]. APC knock-out mice had double the latency to goal, committing three times as many errors, and took less efficient routes to the goal compared to wild-type mice [8]. In this in vivo model, APC loss lead to excessive B-catenin and *Wnt* signal transduction in the forebrain as well as cognitive impairments and an autistic-like presentation [8]. Similarly, Koshimizu et al. had reported that the APC gene plays a role in locomotion and working memory performance. These investigators reported decreased locomotor activity and age-dependent working memory deficits using a heterozygous APC knockout mice model [26]. Hence, our clinical observations of significantly lower performance on IQ and a variety of specific neurocognitive functions among patients with FAP as compared to controls may result from defective APC protein in the synaptic complex in the central nervous system.

Most mutations found in patients with FAP represent truncating mutations of the APC gene [1] and clinical presentations typically follow a genotype-phenotype pattern although there are known exceptions [27, 28]. For instance, severe colorectal polyposis (> 1000 adenomas) correlates with mutations between codons 1250 and 1464 [29], while desmoid tumors may be more prevalent in persons with mutations after codon 1444 [30, 31]. In our current study, we examined neurocognitive functions across two genetically defined subgroups including individuals with attenuated-FAP and those with classic FAP as defined by the location of their *APC* mutations. The location of the mutation in the APC gene correlates with the length and function of the APC protein, with less function seen among those with mutations in the classic FAP region. Our exploratory analysis suggests differences in several categories including cognitive efficiency and processing speed, and selected executive functions (set shifting, initiation) with higher performance among patients with attenuated-FAP compared to those with classic FAP.

Evidence for a potential etiologic role of APC protein in neurodevelopmental disorders arises from human studies examining the association of APC gene polymorphisms and autism spectrum disorders [32]. Neurodevelopmental disorders such as autism spectrum disorder, bipolar disorder and schizophrenia may result from dysregulation of *Wnt* signaling [33], which is also seen in patients with FAP. Furthermore, deletions of the APC gene and occurrence of adenomatous polyposis have been reported in patients originally referred for autism [34]. In our Polyposis Registry [1], we have several patients with either mutations and/or deletion of the APC gene who are also diagnosed with autism spectrum disorder. The APC gene has also been claimed to be associated with susceptibility to schizophrenia [10], providing an additional line of evidence for the important role that APC protein may have in the etiology of neurodevelopmental disorders. Our study provides proof-of-concept and preliminary clinical data of the ubiquitous deficit in neurocognitive functions associated with mutations in the APC gene in a genetically defined syndrome.

The pervasive nature of the observed cognitive dysfunction suggests that loss or dysfunction of the APC protein occurs across the cerebral cortex. Moreover, the observed neurocognitive dysfunction followed a genotype-phenotype correlation as occurs with other clinical manifestations in FAP patients. However, as FAP is a chronic debilitating disease that impacts individuals from early adolescence it may negatively affect cognition by altering educational milestones due to medical/surgical management of the condition. We thus reviewed and contrasted the effect on neurocognitive outcomes in two well characterized genetically-driven chronic illnesses, Cystic Fibrosis and Sickle Cell Anemia [35–37]. Results of these studies showed that children with Cystic Fibrosis had general intelligence levels that were comparable to the general population of the same age group [36]. Furthermore, a study on psychological assessment in adults with Cystic Fibrosis could not detect an increase in depressive or anxiety related symptoms in the affected adults [38]. In contrast, studies on the neurocognitive effects in children and adolescents with Sickle Cell Anemia reported learning difficulties [37], need for special education supports [37], lower visual-motor abilities [39], and decreased verbal short-term memory [35]. These neurocognitive effects were associated with the severity of the Sickle Cell Anemia, which suggest the mechanism for the neurocognitive deficits observed is reduced oxygen delivery rather than other confounding social determinants [35, 37]. The mechanism by which APC mutations cause neurocognitive dysfunction results from the major role of the APC protein in the development of the pre-post synaptic complex [2, 3]. Thus, the mechanisms by which the neurocognitive deficits present in both illnesses (FAP vs. Sickle Cell Anemia) differ in etiology. Our study serves as a proof-of-concept for the potential role of APC protein in neurocognition among patients with FAP; however, our study could not fully account for the possible confounding effect on cognition of having a chronic condition, such as school absenteeism, frequent medical visits, among others.

It is also important to explore how patients with FAP might react to the results obtained in this pilot study, by examining the attitudes, emotional impact and perceptions of these individuals regarding their cognitive abilities. While we are clearly cognizant of this being yet another potential worry for patients with FAP and their families, early recognition of potential risk for cognitive difficulties might lay the foundation for earlier intervention and, perhaps, lessening or preventing such difficulties over the life course for individuals with FAP. Studies on the effects of other chronic illnesses affecting children and young adults are needed to fully elucidate if the neurocognitive effects seen in patients with FAP are partly due to a defective/truncated APC protein and not by other confounders. Our proof-of-concept findings would suggest that this is a viable path for further scientific inquiry.

## Conclusion

Our study in a genetically defined hereditary syndrome provides preliminary clinical data supporting an important role of the APC protein in neurocognitive function and of the pervasive neurocognitive dysfunctions that may be associated with mutations in the APC gene. Validation of our current observations in larger ethnically diverse cohorts would support cognitive screening for individuals with FAP to establish therapeutic interventions early in life as done for other neurodevelopmental disorders. Hence, studies examining the role of the APC protein among individuals with other neurodevelopmental conditions that manifest cognitive impairment, such as autism spectrum disorders and schizophrenia [40, 41] are warranted.

## Data Availability

In accordance with guidelines, unidentified data and materials supporting the results reported in the article will be available upon request. The dataset will be available to interpret, replicate and build upon the findings reported in this article.
